# Statins and aspirin in the prevention of cardiovascular disease among HIV-positive patients between controversies and unmet needs: review of the literature and suggestions for a friendly use

**DOI:** 10.1186/s12981-019-0226-2

**Published:** 2019-05-24

**Authors:** P. Maggi, G. V. De Socio, S. Cicalini, M. D’Abbraccio, G. Dettorre, A. Di Biagio, C. Martinelli, G. Nunnari, S. Rusconi, L. Sighinolfi, V. Spagnuolo, N. Squillace

**Affiliations:** 1 Università degli Studi della Campania Luigi Vanvitelli, Naples, Italy; 2 Clinica Malattie Infettive, Azienda Ospedaliero-Universitaria di Perugia, Perugia, Italy; 3 INMI L. Spallanzani, Rome, Italy; 4 UOC. di Immunodeficienze e Malattie Infettive di Genere, P.O. “D. Cotugno” - AORN Dei Colli, Naples, Italy; 5 Clinica Malattie Infettive. Policlinico Umberto I, Rome, Italy; 6 Clinica Malattie Infettive IRCCS AOU San Martino-IST, Genoa, Italy; 7 SOD Malattie Infettive e Tropicali AOU Careggi, Florence, Italy; 8 Clinica Malattie Infettive Policlinico, Messina, Italy; 9 Divisione Clinicizzata AOU L. Sacco, Milan, Italy; 10 U.O.Malattie Infettive AOU Ferrara, Ferrara, Italy; 11 Dipartimento Malattie, Infettive Ospedale S. Raffaele, Milan, Italy; 12 Divisione Malattie Infettive AO S. Gerardo, Monza, Italy

**Keywords:** Statins, Aspirin, HIV, Cardiovascular disease, Antiplatelet agents, Oral anticoagulants

## Abstract

**Background:**

As in non-infected subjects, statins and aspirin have a pivotal preventive role in reducing the cardiovascular related morbidity and mortality in HIV infected patients. The persistence of immune activation in these subjects, could contribute to accelerate atherosclerosis, therefore, these treatments that reduce inflammation could provide additional cardiovascular protection. However the current guidelines for the use of these drugs in general population are dissimilar, with important differences between American and European ones. Aim of the present position paper is to provide recommendations aimed to overcome the actual differences and limitations among the current ones and to adapt them to the needs of HIV infected patients.

**Results:**

We propose to adopt the new ACC/AHA guidelines, simple to use and cost effective, to use the ASCVD score that seems to estimate more accurately the cardiovascular risk among these patients. We suggest to start statin therapy in all patients with a calculated 10-year risk of a cardiovascular event of 10% or greater. Rosuvastatin and atorvastatin should be preferred. LDL-C target may be adopted. Aspirin should be always associated with a statin, in secondary prevention, while in primary prevention it should be reserved only to patients with ≥ 20% 10-year risk particularly adherent to treatments, and with low risk of bleeding. We suggest to start with a dose of 100 mg/day. Finally, management of antiplatelet agents or novel oral anticoagulants may include selecting antiretrovirals with a lower potential for drug interactions or choosing agents least likely to interact with antiretrovirals.

**Conclusions:**

As demonstrated in surveys, HIV physicians are generally highly committed regarding CVD and autonomous in prescribing statins and ASA. Consequently, in the light of the previously discussed discrepancies among the different guidelines and of the incomplete indications regarding HIV-positive persons, the present suggestions could overcome the actual differences and limitations among the current ones.

## Background

The introduction of combined antiretroviral therapy (cART) has greatly reduced the risk of death from AIDS-related causes leading to a considerable increase in the life expectancy of people living with HIV (PLWHIV). Actually, the main factor influencing the prognosis of PLWHIV is the onset of non-AIDS-defining events as liver disease, renal impairment, cancer, and cardiovascular disease (CVD). In particular, results from several studies have suggested that PLWHIV have an increased risk of CVD, especially coronary heart disease, compared with people not infected with HIV [1–10]. Overall the incidence of CVD in HIV is relatively low, but it is approximately 1.5–2-fold higher than that seen in age-matched HIV-uninfected individuals. PLWHIV are exposed both to an increased prevalence of traditional CVD risk factors, and to HIV-specific mechanisms such as inflammation [1–3]. The reasons of the increased risk remains not completely understood, however, endothelial activation due to the chronic inflammation seems to play a pivotal role in CVD events [4]. In fact, a body of evidence documented that in HIV patients atherosclerosis is accelerated and chronic inflammatory processes are activated [5, 6]. The early and continuous use of current cART, with fewer metabolic effects, minimizes the risk of myocardial infarction (MI) by maintaining viral suppression and decreasing immune activation. Even with cART however, immune activation persists in PLWHIV and could contribute to accelerate atherosclerosis [6–9]. Therefore, treatments that safely reduce inflammation in PLWHIV could provide additional cardiovascular protection alongside treatment of both traditional and non-traditional risk factors. As in non-infected subjects statins and aspirin have a pivotal preventive role in reducing the CV related morbidity and mortality in HIV infected patients. Below we focused the actual unmet needs in the use of statins and aspirin in PLWH and indicate our suggestions to overcome the discrepancies and incompleteness of the current guidelines.

## Controversies between guidelines

About 30 years ago statins inaugurated the era of lipid lowering therapy as the most effective way to reduce the risk of atherosclerotic CVD (ASCVD). More recently, it has been demonstrated that statins, through their HMG-CoA reductase inhibitor activity, have pleiotropic immunomodulatory properties that contribute to their benefit in atherosclerosis beyond lipid lowering [11, 12]. However the current guidelines for the use of these drugs in general population are dissimilar, with important differences between American [13] and European ones [14]. The European Society of Cardiology (ESC) and the European Atherosclerosis Society (EAS) guidelines for the management of dyslipidaemia and the use of statins in CVD prevention suggest to evaluate the total CV risk of the subjects by using European SCORE tables, identify the LDL-C target for that risk level, calculate the percentage reduction of LDL-C required to achieve that goal, and choose a statin that, on average, can provide this reduction.

Differently, the American College of Cardiology/American Heart Association (ACC/AHA) identifies four statin benefit groups in which the potential for an ASCVD risk reduction benefit clearly exceeds the potential for adverse effects (1—individuals with clinical ASCVD; 2—individuals with primary elevations of LDL-C ≥ 190 mg/dL; 3—individuals aged between 40 and 75 years with diabetes and LDL-C 70–189 mg/dL; 4—individuals without clinical ASCVD or diabetes who are 40 to 75 years of age with LDL-C 70–189 mg/dL and an estimated 10-year ASCVD risk of 7.5% or higher), identifies high-intensity and moderate-intensity statin therapy for use in secondary and primary prevention and suggest the appropriate intensity of statin therapy to reduce ASCVD risk in those most likely to benefit. On the other hand, this Expert Panel was unable to find evidence to support continued use of specific LDL-C and/or non-HDL-C treatment targets. Finally, this guideline recommends use of the new Pooled Cohort Equations to estimate 10-year ASCVD risk.

Some European Authors took position against ACC/AHA guidelines, objecting that if generally adopted, will result in an increase in the number of patients treated, potentially at considerable cost. Moreover, the new pooled mixed cohorts equation used to assess ASCVD risk has been validated in an American population, different from European countries and requires more careful evaluation if applied in other contexts [15]. In summary, the debate between American and European guidelines is still open.

More recently [16], the US Preventive Services Task Force (USPSTF) recommends that adults without a history of CVD (i.e., symptomatic coronary artery disease or ischemic stroke) use a low- to moderate-dose statin for the prevention of CVD events and mortality when all of the following criteria are met: 1—they are aged 40 to 75 years; 2—they have 1 or more CVD risk factors (i.e., dyslipidemia, diabetes, hypertension, or smoking); and 3—they have a calculated 10-year risk of a cardiovascular event of 10% or greater. The treatment is optional if the calculated 10-year risk is between 7.5 and 10%.

On the other side, aspirin (ASA) remains one of the most extensively studied cardiovascular medications in the history of medicine. The drug reduces the incidence of MI, stroke or vascular death in patients with vascular disease via his antiplatelet activity. However, despite multiple, well-designed, large randomized controlled trials evaluating the potential of aspirin to prevent cardiovascular events in individuals without known CVD, the role of aspirin in primary prevention is currently unclear. The initial aspirin trials included largely low-risk individuals with primary outcomes mostly focused on MI and stroke, and showed a significant reduction in these CVD outcomes, especially MI. In the following years, trials have focused on older, higher CVD risk populations with high rates of lipid-lowering and antihypertensive medications use. These studies have used broader CVD outcomes as their primary end-points and have failed to show a significant benefit of aspirin therapy in primary prevention. The exact reasons for the lack of efficacy in these recent trials are unclear but might be related to low rate of atherothrombotic events relative to other CVD events in the populations studied [17]. Recently, a number of major trials addressed the problem of aspirin in primary prevention. In ASCEND trial [18], aspirin prevented serious vascular events in persons who had diabetes and no evident cardiovascular disease at trial entry, but it also caused major bleeding events. The absolute benefits were largely counterbalanced by the bleeding hazard. Moreover, in ASPREE trial [19] the use of low-dose aspirin as a primary prevention strategy in older adults resulted in a significantly higher risk of major hemorrhage and did not result in a significantly lower risk of cardiovascular disease than placebo. ASPREE also showed that aspirin use in healthy elderly persons did not prolong disability-free survival over a period of 5 years but led to a higher rate of major hemorrhage than placebo [20]. Unexpectedly, in the same trial, higher all-cause mortality was observed among apparently healthy older adults who received daily aspirin than among those who received placebo and was attributed primarily to cancer-related death. The same Authors state that this result was unexpected and should be interpreted with caution [21]. In ARRIVE trial [22], that explored the use of aspirin to reduce risk of initial vascular events in patients at moderate risk of cardiovascular disease, the event rate was much lower than expected, which is probably reflective of contemporary risk management strategies, making the study more representative of a low-risk population.

Differently, the evidence supporting aspirin for secondary CV prevention in the general population is stronger: in high risk patients ASA reduces the yearly risk of serious vascular events (non-fatal MI, non-fatal stroke, or vascular death) by about a quarter [23]. However, nowadays aspirin is recommended in secondary CV prevention as well for men age 45 to 79 years when the potential benefit due to a reduction in MI outweighs the potential harm due to an increase in gastrointestinal hemorrhage and for women age 55 to 79 years when the potential benefit of a reduction in ischemic strokes outweighs the potential harm of an increase in gastrointestinal hemorrhage [24–26].

## The unmet needs

As discussed above, the effect of statins and ASA in preventing CVD is linked to their anti-inflammatory activity on vessels; consequently there is a stronger rationale in their use among PLWHIV with respect to the general population. In spite of this, the guidelines for PLWHIV, reflecting the challenges of the guidelines for general population, result sometimes incomplete. In fact, the European AIDS Clinical Society (EACS) version 8, recommends the use of statin in patients with established CVD or type 2 diabetes or 10-year CVD risk ≥ 10% irrespective of lipid levels. Similarly, ASA is recommended in patients with previous CVD or aged ≥ 50 years and at high (≥ 20%) 10-year CVD risk [27]. Also for these reasons, some reports underline the underuse of aspirin in HIV patients and few data are available on use of statins and ASA in such a setting in clinical practice [28, 29]. Also, a recent Italian study evidences that the prescription of statins and aspirin in HIV infected patients remains largely suboptimal, as only about 50% of patients requiring their use are properly treated [30].

A recent survey among Italian HIV specialists conducted by administering a questionnaire aimed at investigating the utilization of statins and ASA, the use of guidelines and scores and the management of interactions ha provided homogeneous results in the different geographic areas [31]. The majority directly prescribe statins and 43% of them prescribe aspirin; most of them follows guidelines and utilizes scores to calculate the CV risk. The survey demonstrates the high attention of HIV physicians regarding CVD, their commitment and autonomy in prescribing statins and ASA.

Consequently, in the light of the previously discussed discrepancies among the different guidelines and of the incomplete indications regarding HIV-positive persons, there is a strong rationale to generate specific guidelines for HIV infected patients able to overcome the actual differences and limitations among the current ones.

### (A) Statins

As we illustrated previously, studies regarding statins have been performed in the US, and therefore their results may not necessarily apply to other than US settings; consequently there is still a need to validate the ACC/AHA ASCVD score at least in the European HIV adult population. On the other hand, the new ACC/AHA guidelines are very simple to use being based only on the classification of the patients in one of the four statin benefit groups and having eliminated the necessity of the up-titration of the drugs to reach the LDL-C and/or non-HDL-C treatment target. As seen previously, some European authors object that ACC/AHA guidelines, based on a “fire and forget” strategy, will result in an increase in the number of patients treated with statins, potentially at considerable cost. Actually, the anti-inflammatory properties of these drugs, beyond lipid lowering, could be beneficial in the light of the chronic inflammation condition of PLWH. In fact, a recent study observed that statin use was associated with significantly lower hazard of dying in these patients who were being effectively treated with cART as determined by virological suppression [32]. Other data on this topic will be generated by REPRIEVE (Randomized Trial to Prevent Vascular Events in HIV), a large, multicenter study funded by the American National Institutes of Health, testing whether pitavastatin, a newer statin that does not have substantial interactions with antiretroviral drugs, can prevent vascular events over time among HIV-infected individuals who do not have known CVD. This study is now open to enrollment at sites throughout the United States and abroad and will hopefully provide definitive data on the effects of statin use among PLWH [33].

Consequently, we believe that ACC/AHA guidelines simple to use, and cost effective, should be adopted in the management of PLWH and use of statins, when indicated, should be encouraged.

#### (1) Which algorithm to adopt to estimate the CV risk of PLWH?

European Authors object that the new pooled mixed cohorts equation used to assess ASCVD risk in the 2013 ACC/AHA guidelines (PCE) has been validated in an American population different from European countries and requires more careful evaluation if applied in other contexts. Actually, some studies suggest that PCE score could better estimate the CV risk among PLWH; in fact, using data from 2283 HIV-infected adults from the HIV Outpatient Study (HOPS), Thompson-Paul [34] assessed performance of three CVD prediction models developed for general populations (Framingham general cardiovascular Risk Score [FRS], ACC/AHA PCE, and Systematic COronary Risk Evaluation [SCORE] high-risk equation), and the Data Collection on Adverse Effects of Anti-HIV Drugs (D:A:D) study equation, a model developed in HIV-infected persons. The results evidenced that only the FRS accurately estimated risk of CVD events, while ACC/AHA PCE and D:A:D underestimated risk. In another study, Crane and coll [35] developed a state-of-the-art screening algorithm and central adjudication protocol for the validation of incident acute myocardial infarction (AMI) in the CFAR Network of Integrated Clinical Systems (CNICS), which harmonizes comprehensive clinical data on PLWH in routine care at multiple US sites. Among PLWH enrolled between 1996 and 2014, they compared the performance of 3 CVD risk scores developed in the general population: FRS, ATP-III, and 2013 ACC/AHA PCE, and one developed for use in PLWH: D:A:D using area under the curve (AUC). ACC/AHA PCE had a significantly better AUC than other scores for all AMI and for Type 2 AMI including the DAD AUC (p < 0.001), and was not inferior to the other AUCs for Type 1 acute AMI. In summary, the current prediction models seem to underestimate the CV risk, probably because PLWH have additive risk factors, such as the state of the infection and the inflammatory condition, difficult to measure. However, the ACC/AHA PCE seems to estimate more accurately the CV risk among PLWH. In addition, ACC/AHA PCE is a well validated score deriving from FRS that have a major historical background, its calculation is simple, adequate for non specialist physician and evaluates also non-fatal events.

#### (2) At what percentage of risk should we start a statin therapy?

We suggest to start statin therapy in all patients with a calculated 10-year risk of a cardiovascular event of 10% or greater. Considering the increase of pill burden, the treatment is optional if the calculated 10-year risk is between 7.5 and 10%.

#### (3) Which statin to use?

Regarding statins, the ACC/AHA guidelines suggest to chose the appropriate intensity of statin therapy to reduce CVD risk considering that, a therapy with a high intensity statin daily lowers LDL-C on average by approximately ≥ 50%, a moderate intensity statin therapy lowers LDL-C on average by 30% to < 50%, and a low intensity statin therapy lowers LDL-C on average by < 30%. When we prescribe a statin to PLWH the problem of drug–drug interactions must be attentively evaluated. In particular, as suggested by EACS Guidelines [27], in patients treated with protease inhibitors/ritonavir, simvastatin is contraindicated and atorvastatin and rosuvastatin should be started with low dose; rosuvastatin should be started with low dose also in patients treated with non-nucleoside. Finally, when used with darunavir/ritonavir, also pravastatin should be started with lower dose. However, rosuvastatin and atorvastatin, when indicated, should be preferred because a body of evidence demonstrate their efficacy and safety in PLWH [36]. Pitavastatin has as yet no morbidity/mortality trial data to support its use but may have advantages of fewer drug–drug interactions, more HDL-C increase and less adverse glucose effect than other statins [28, 36].

#### (4) Should we use statins in patients with subclinical atherosclerosis?

A body of evidence demonstrates that statin therapy is associated with a favorable decrease in intima media thickness of common carotid arteries both in free population [37] and among PLWHIV [38, 39]. In particular, in a randomised, double-blind, placebo-controlled trial atorvastatin, statin therapy reduced non-calcified plaque volume and high-risk coronary plaque features in HIV-infected patients in a study based on fluorodeoxyglucose-PET [38]. In another, rosuvastatin effectively lowers LDL-C and appears to substantially slow progression of common carotid intima media thickness in patients with treated HIV infection [39]. Consequently, we encourage the use of a high intensity statin (atorvastatin or rosuvastatin) in PLWH with a subclinical atherosclerosis regardless the levels of LDL-C or the calculated 10-year risk.

#### (5) Should we use LDL-C target in PLWH?

ACC/AHA guidelines [13] do not recommend LDL-C target for statin therapy, in contrast to the European ones [14] and EACS guidelines [27]. In the general population, target levels for statin therapy are extrapolated from several clinical trials [40]. Specific data regarding PLWH are unavailable. However PLWH usually perform laboratory controls every 6–12 months including LDL-C levels, consequently the efficacy of the statin therapy in our patients is constantly monitored also the potential drug–drug interaction between antiretrovirals and statins imposes periodic controls of the LDL-C levels.

Consequently, we believe that LDL-C target may be adopted in the management of PLWH as indicated by EACS guidelines, as a part of the routine controls of the patients.

### (B) Aspirin

#### (1) Should we use aspirin in primary CV prevention?

As seen before, the evidence supporting ASA for primary CV prevention are inconsistent. 2019 ACC/AHA guidelines allow aspirin in primary prevention, based on a weak level of evidence (IIb), and suggest that low-dose aspirin (75–100 mg orally daily) might be considered for the primary prevention of ASCVD among select adults 40 to 70 years of age who are at higher ASCVD risk but not at increased bleeding risk [41]. As a matter of fact, these suggestions do not allow an individualised risk benefit assessment for aspirin use in the people at highest CVD risk with the weakest level of recommendation. However, the EACS guidelines still does not incorporate findings from recent trials and ACC/AHA guidelines in people at higher CVD risk, and still recommends the use of aspirin 75–150 mg in patients aged ≥ 50 years and at high (≥ 20%) 10-year CVD risk [27].

We feel that, in this setting, the balance between cost and benefit should be attentively evaluated considering that, if on one hand there are no strong evidences of a benefit, on the other hand the addition of another daily pill, increasing the pill burden could determine a decrease of the adherence to therapy included the antiretroviral one. Moreover we should consider the risk of bleeding, especially in patients with liver disease and uncontrolled hypertension. These patients should be treated with a statin as suggested by the ACC/AHA guidelines. Consequently, the addition of aspirin should be reserved only to patients with ≥ 20% 10-year CVD risk that are particularly adherent to treatments, and with low risk of bleeding.

#### (2) Should we use aspirin in secondary CV prevention?

In line with the previously cited guidelines for general population and for PLWH, aspirin should be always used in secondary CV prevention, in association with a statin. In the light of the reported underutilization of ASA and statins in clinical practice [28–30] the HIV physicians should strongly encourage the prescription and the adherence to these drugs in their patients.

#### (3) With which dose of aspirin should we use in PLWH?

Considering the potential side effects and the dosage of the most common commercially available preparations of the drug, we suggest to start with a daily dose of 100 mg.

#### Other potential non-cardiovascular benefits of aspirin

In general population there is increasing evidence for a chemopreventive effect of low-dose aspirin against colorectal (and other) cancer [42]. Even if the literature in HIV patients is, at present, scarce and inconsistent, this potential additive benefit could be considered in prescribing aspirin in HIV patients, considering that they are more prone to chronic inflammation and non-AIDS-defining cancers respect to general population [43].

### (C) Antiplatelet agents or novel oral anticoagulants

#### How to manage the co-medication with antiplatelet agents or novel oral anticoagulants?

Not infrequently in case of CVD, specialists prescribe to PLWH other drugs, such as antiplatelet agents or novel oral anticoagulants. Recently, potential drug interactions between antiretroviral therapy and these molecules have been demonstrated. In fact, in vivo interaction have been documented between ritonavir and prasugrel, efavirenz and clopidogrel, nevirapine and rivaroxaban. Consequently, these interactions should be attentively avoided when antiplatelet agents or novel oral anticoagulants are prescribed. Clinicians should consider that with protease inhibitors (PI) or cobicistat (COBI), prasugrel do not appear to have clinically significant interactions. Tigacrelor should not be coadministered with PI or COBI. Non-nucleoside reverse transcriptase inhibitors have a low potential for interactions with prasugrel and dabigatran. Clinically significant drug interactions are unlikely to occur between antiplatelet agents or novel oral anticoagulants and nucleoside reverse transcriptase inhibitors, raltegravir, dolutegravir, or maraviroc. Management of these drugs may include selecting antiretrovirals with a lower potential for drug interactions or choosing antiplatelet agents or novel oral anticoagulants least likely to interact with antiretrovirals [44].

## Conclusions

To overcome the actual differences and limitations among the current guidelines for the use of statins and aspirin, and to adapt them to the needs of HIV infected persons, we propose to adopt the new ACC/AHA guidelines, and to use the ASCVD score that seems to estimate more accurately the cardiovascular risk among these patients. We suggest to start statin therapy in all patients with a calculated 10-year risk of a cardiovascular event of 10% or greater, preferring rosuvastatin and atorvastatin. Aspirin should be always associated with a statin, in secondary prevention, while in primary prevention it should be reserved only to patients with ≥ 20% 10-year risk particularly adherent to treatments, and with low risk of bleeding, starting with a dose of 100 mg/day. Finally, management of antiplatelet agents or novel oral anticoagulants may include selecting antiretrovirals with a lower potential for drug interactions or choosing agents least likely to interact with antiretrovirals. Our positions have been summarized as take home messages in Table 1.

**Table 1:** Take home messages

(a) Statins	(1) Which guidelines to adopt between American and European ones?
	ACC/AHA guidelines, being simple to use and cost effective, should be adopted in the management of PLWH and use of statins, when indicated, should be encouraged
	(2) Which algorithm to adopt to estimate the CV risk of PLWH?
	The current prediction models seem to underestimate the CV risk, However, the ACC/AHA PCE score seems to estimate more accurately the CV risk among PLWH. In addition ACC/AHA PCE is well validated, its calculation is simple, and evaluates also non-fatal events
	(3) At what percentage of risk should we start a statin therapy?
	We suggest to start statin therapy in all patients with a calculated 10-year risk of a cardiovascular event of 10% or greater. Considering the increase of pill burden, the treatment is optional if the calculated 10-year risk is between 7.5 and 10%
	(4) Which statin to use?
	In line with ACC/AHA guidelines, we suggest to chose the appropriate intensity of statin therapy to lower LDL-c by the requested percentage. When a statin is prescribed to PLWH the problem of drug–drug interactions must be attentively evaluated consulting the current guidelines for the use of antiretrovirals
	(5) Should we use statins in patients with subclinical atherosclerosis?
	We encourage the use of a high intensity statin (atorvastatin or rosuvastatin) in PLWH with a subclinical atherosclerosis
(b) Aspirin	(1) Should we use aspirin in primary CV prevention?
	The balance between cost and benefit should be attentively evaluated considering that there are no strong evidences of a benefit, and that the increasing of the pill burden could determine a decrease of the adherence to antiretroviral therapy. Moreover the risk of bleeding should be considered, especially in patients with liver disease and uncontrolled hypertension. Consequently the addition of aspirin should be reserved only to patients with ≥ 20% 10-year CVD risk that are particularly adherent to treatments, and with low risk of bleeding
	(2) Should we use aspirin in secondary CV prevention?
	Aspirin should be always used in secondary CV prevention, in association with a statin. The HIV physicians should strongly encourage the prescription and the adherence to these drugs. We suggest to start with a dose of 100 mg/day
(c) Antiplatelet agents or novel oral anticoagulants	(1) How to manage the co-medication with antiplatelet agents or novel oral anticoagulants?
	Potential drug interactions between antiretroviral therapy and these molecules have been demonstrated. Consequently, management of these drugs may include selecting antiretrovirals with a lower potential for drug interactions or choosing antiplatelet agents or novel oral anticoagulants least likely to interact with antiretrovirals

## Data Availability

Not applicable.
